# Osteocalcin induces phosphorylation of FOXO1 in human beta-cells and restores insulin expression under hyperglycemic conditions

**DOI:** 10.21203/rs.3.rs-6474216/v1

**Published:** 2025-06-13

**Authors:** Shubhashish Sarkar, A. Osama Gaber, Christine A. Beamish, Omaima M. Sabek

**Affiliations:** 1 Department of Surgery, the Houston Methodist Hospital, Houston, TX; 2 Department of Cell and Microbiology Biology, Weill Cornell Medical Biology, NY

**Keywords:** Pancreatic islet, osteocalcin, human, beta cell, FOXO1

## Abstract

Forkhead box O1 (FOXO1) is a key transcription factor that plays an important role in pancreatic β-cell compensation under physiological and pathological conditions and serves as a key regulator of glucose homeostasis. While FOXO1 expression in osteoblasts contributes to glucose maintenance through regulating osteocalcin, interestingly, osteocalcin acts directly on β-cells by regulating PDX1 and insulin expression. Here, we investigate the effect of osteocalcin on the FOXO1 expression in human pancreatic β-cells.

In a human β-cell line and pancreatic islets, the fate of FOXO1 binding to the PDX1 promoter was investigated after osteocalcin treatment, with or without AKT inhibition. Furthermore, we investigated the effect of osteocalcin on PDX1 and insulin gene expression as well as the subcellular localization of FOXO1 and PDX1 in human islets.

The data show that osteocalcin treatment increased the amount of phosphorylated FOXO1-S256 via AKT in human islet from high BMI donor. Moreover, human islets from donors with and without diabetes treated with osteocalcin showed a reduced nuclear FOXO1 and an increase in nuclear PDX1.

In a human β-cell line and pancreatic islets, osteocalcin increases insulin and PDX1 expression following phosphorylation-dependent ubiquitination and degradation of FOXO1 via the protein kinase B pathway.

## Introduction

Prototypical and pathological characteristics of type 2 diabetes mellitus (T2DM) such as peripheral insulin resistance and impaired β-cell compensation predisposes the β-cell to failure via impaired FOXO1 phosphorylation ^1^. Hyperglycemia is associated with rapid and marked dephosphorylation and activation of FOXO1 ^1^. Chronic hyperglycemia and impaired FOXO1 phosphorylation lead to the reduction of key β-cell transcription factors such as PDX1 ^2^. FOXO1 and PDX1 exhibit mutually exclusive patterns of nuclear localization in β-cells, and constitutive nuclear expression of a mutant FOXO1 is associated with loss of PDX1 expression ^3^. Furthermore, PDX1 plays a significant role in the maturation and identity perseveration of β-cells as it controls the activation of insulin and other genes responsible for glucose sensing and metabolism ^4^. Beta-cells with genetically deleted PDX1 acquire ultrastructural and physiological features of α-cells, indicating that a cellular reprogramming or dedifferentiation occurred, comparable to the dedifferentiation and loss of β-cell function in islets from patients with diabetes ^5^. Dedifferentiated human β-cells in islets from donors with diabetes and prediabetes have been identified as hormone- or transcription factor-negative β-cells ^6^, double-positive β-cells ^6,7^, and/or degranulated β-cells ^8^.

The hormone osteocalcin (OC) is secreted from osteoblasts into blood circulation ^9^. Synthesized OC in the osteoblast can become gamma (γ)-carboxylated, which has a high affinity for bone, or it can become decarboxylated, which reduces its affinity for bone and triggers the release of uncarboxylated OC into the blood circulation. Although OC was originally thought to play a major role in osteoblastic bone formation, recent murine and *in vitro* studies have also revealed that the uncarboxylated form of OC regulates glucose metabolism and insulin production in pancreatic islets, with subsequent reduction of blood glucose levels ^10-13^, and increased human insulin and c-peptide was released when islets were exposed to OC in mouse transplant experiments ^13^. We have shown previously that OC protects against metabolic dysfunction-associated steatohepatitis and islet identity loss in rodent models of metabolic syndrome ^10,12^. Others have reported in high-fat-diet models that OC-treated mice showed reduced body weight and fat pad gain, and improved insulin sensitivity and energy expenditure versus control-treated mice ^14^. An inverse relationship between uncarboxylated OC and fasting glucose and insulin resistance was found in obese women without diabetes ^15^, and elsewhere, age, percent fat, high-density lipoprotein cholesterol, fasting plasma glucose, and fasting serum insulin were independently associated with OC in men ^16^. Moreover, total OC was found to be inversely related to BMI and waist circumference in young adults, which suggests a role for OC in the development of insulin resistance concomitant with aging ^17^. The same inverse relationship between total serum OC and glucose homeostasis has been extensively documented in adults with pre-diabetes and T2DM ^18-22^. Indeed, intermittent injection of OC was shown to improve hyperglycemia and prevent T2DM in mice ^23^.

The mechanism of glucose regulation by OC involves binding to and activating GPRC6A, a class-C G-protein coupled receptor, in target tissues ^23-25^. Structural modeling suggests sites in the heptahelical domain of GPRC6A that bind OC, as well as showing where an OC-derived hexapeptide from the peptide C-terminus (OC-6aa-C) docks with GPRC6A ^26^. Interestingly, GPRC6A activates the same signaling pathways that are targeted by OC, including extra-cellular signal regulated kinases (ERK), and phospho-inositide 3-kinase/protein kinase B (PI3K/AKT) ^26-29^. However, the effect of OC on the phosphorylation of FOXO1 and the expression of PDX1 in human pancreatic β-cells remains to be explored. Here, we show through cell signaling that FOXO1 is phosphorylated at serine (S)256 via activation of protein kinase B (AKT) by OC, resulting in FOXO1 nuclear export and/or degradation, allowing the expression of PDX1, and hence restoring insulin gene expression.

## Materials And Methods

### Human pancreatic islets

Human pancreatic islets and/or other resources were provided by the NIDDK-funded Integrated Islet Distribution Program (IIDP) (RRID:SCR_014387) at City of Hope, NIH Grant # 2UC4DK098085.

All human islets utilized were from donors with high BMI (> 28 kg/m2; mean ± SEM 36.7± 2.2), three without diabetes (HbA1_c_ < 6.4%) and two with diagnosed T2DM (HbA1_c_ > 6.4%); donor demographics are shown in Table 1.

### Islet culture

Aliquots from human islet isolations were cultured in Memphis (serum-free) medium supplemented with 10 U/mL heparin and 10 mM niacin in 5.5 mmol/L glucose as previously described ^30^. Islet culture media was changed at day 1 after plating and twice weekly thereafter during the culture period. The islets were cultured for 1–3 days before treatment initiation to allow for sterility and viability testing. Islets were compared with those cultured in media supplemented with 4.5 ng/ml OC for 7 days; OC dose response in human islets was reported by us previously ^13^.

### Cell culture

Human embryonic kidney (HEK)293 cells were purchased from ATCC (Manassas, VA, USA) and maintained in DMEM medium (Gibco,11966025) supplemented with 10% fetal bovine serum, 100 units of penicillin and 100 g/ml streptomycin (Gibco, 15140122) at 37°C in a 5% CO_2_ incubator. The pancreatic electrofusion hybrid human β-cell line 1.2B4 (Sigma, 10070103 ^10^), was maintained in RPMI 1640 medium (Corning 10-104-CV). Both contain 2 mM glutamine with the same supplements supplemented with 10% fetal bovine serum, 100 units of penicillin and 100 g/ml streptomycin (Gibco, 15140122).

For treatments, cells were seeded in regular culture medium as described above. After overnight culture, cells were starved for 24h in DMEM medium the absence of glucose and serum. The cells were then treated in DMEM medium with 4.5 ng/ml OC ^10,13^ or other specific inhibitors in 0.1% FBS at a glucose concentration of 16.7 mM for 3, 6 or 12h. Specific inhibitors included the AKT inhibitor (Sigma, 124005; 10 μM) and the proteasomal inhibitor MG132 (Sigma, M8699; 20 μM ). After treatment, cells were collected with 0.25% trypsin (Fisher Scientific, 25200072), washed 3 times with cold 25mM Tris-HCl (pH 7.4) buffer and froze at −80°C for lysate preparation. For the chromosome immunoprecipitation (ChIP) assay using human islets, media and treatments were changed every 48h, and islets were cultured for 7d total.

### Western blot analysis

Cell extracts were made in 1X cell lysis buffer (Cell Signaling Technology [CST], 9803) supplemented with phenylmethyl sulfonyl fluoride (PMSF) and protease inhibitors (Sigma, P8340). cells were incubated with cell lysis buffer and incubated on ice for 30 min, followed by homogenization in Precellys bead homogenizer (Bertin Corp. Rockville, USA). Homogenized cells were centrifuged at 10,000 RPM for 10 mins and cell supernatant was collected for protein estimation using a Bradford assay. Approximately 30-50 μg protein extract was used for western blot analysis after resolving it in SDS-PAGE. Proteins were transferred to nitrocellulose membranes using semi-dry transfer apparatus (Bio-Rad) for 50 min at 150 mAMP. Membranes were blocked in 5% milk (Sigma, M7409) followed by incubation with specific antibodies at 4°C overnight. Membranes were washed 3x with Tris-HCl + 0.1% Tween 20 buffer for 30 min. Bands were visualized by incubating with ECL reagent (Fisher Scientific, 34577) and exposed to X-ray film (Fisher Scientific, PI34091). The film was scanned, and band density was analyzed by Image-J software. Antibodies used for western blot analysis were FOXO1 Total (CST 2880), phosphorylated FOXO1-S256 (CST 9461), total AKT (CST 4685S), phosphorylated AKT (Ser473) (D9E) CST 4060), HA (6E2, CST2367), PDX1 (Abcam ab219207), and β-Actin (Sigma A3854).

### Ubiquitination Assay

To detect ubiquitination of FOXO1 in HEK293 cells, plasmids of ubiquitin (HA Tag) (GenScript) and FOXO1 (DYK tag) were co-transfected using Turbofectin (OriGene; SKU TF81001) transfection reagent. reagent. Approximately 36h later, cells were starved for 12h and treated for 24h with OC, MG132, or both at a glucose concentration of 16.7 mM. After 24h, cells were harvested and FOXO1 was immunoprecipitated using a DYK tag antibody (CST;14793S) as described by Sarkar *et al* ^31^. The immunoprecipitated complex was resolved by 10% SDS-PAGE, transferred to a nitrocellulose membrane, and incubated with HA Tag Ab (CST; 2367S) to detect ubiquitinated FOXO1. The blots thereafter were treated similarly as western blots described above.

### RNA isolation and RT-PCR

Following treatment, cells were washed in cold PBS and harvested by trypsinization. Total RNA was extracted by QIAwave^®^ RNA Mini Kit (Qiagen,74534). Aliquots of 1μg (1.2B4 cells) or 0.3 μg (human donor islet) of total RNA was reverse transcribed by reverse transcriptase (RT) (NEB, LunaScript RT Master Mix Kit (Primer-free), E3025S) The cDNA obtained was diluted 1:10 in water and was used for PCR using specific primers (Table 2). Agarose gels (1%) were stained with ethidium bromide and scanned by a BioRad Imager. Images were analyzed by Image-J by normalizing the experimental band density with β-actin as an internal control.

### Chromatin Immuno-precipitation assay (ChIP)

Chromatin immunoprecipitation (ChIP) assays were performed as described previously ^32,33^. In brief, after treatment β-cells were fixed in 1% formaldehyde for 10 min to crosslink DNA to bound proteins, followed by the addition of 0.125 mol/L glycine to quench the reaction. cells were washed with cold PBS, pelleted by centrifugation, and resuspended in ChIP sonication buffer (Cell Signaling Tech. Cat# 9803). cells were kept in ice cold water and sonicated using Fisher Scientific (Model FB120) at a power setting of 4 for 30 x 10sec pulses with 1-2 min on ice for cooling time in between pulses for a total of 15 pulses. The fixed cells were sonicated and 600-700bp fragments were used for immunoprecipitation. The cross-linked and fragmented DNA was immunoprecipitated by FOXO1 antibody (1-2 μg of purified antibody) overnight with constant agitation in a rotator. In parallel, heat-killed FOXO1 antibody was used as negative control under equivalent conditions. After overnight incubation, Protein A/G Sepharose beads (Sigma) preabsorbed with Herring sperm DNA (100μg/ml; Sigma, D3159) were added to the DNA-antibody mixture and incubated for an additional 4h at 4°C under constant agitation in a rotator. The Sepharose beads were centrifuged, the supernatant separated, and beads washed with low-salt buffer (0.1%SDS, 0.1% Triton X100, 150mM NaCl and 20mM Tris-Cl pH8.0) followed by elution of the DNA in high-salt buffer (0.1%SDS, 0.1% Triton X100, 500mM NaCl and 20mM Tris-Cl pH8.0). The DNA was precipitated using high-salt buffer as described previously ^34^. The precipitated DNA was further purified using a spin column and used for PCR of the immunoprecipitated DNA with specific primers for sites at −2077 bp and −601 bp of the PDX1 promoter. The primer sequences are shown in Table 2.

### Ethical approvals

Animal experiments were performed in compliance with the ARRIVE guidelines (https://arriveguidelines.org). All animal protocols were approved by the Institutional Animal Care and Usage Committee (IACUC) at the Houston Methodist Research Institute and in accordance with the National Institute of Health Guide for the Care and Use of Laboratory Animals. Human islets were used with approval from the Institutional Review Board of the Houston Methodist Research Institute.

### Human islet transplantation

After 7d of culture, aliquots of 750-1000 IEQ human islets were transplanted under the left kidney capsule of 8–10-week-old female athymic nude *Foxn1nu* mice (n = 4-5 mice/treatment). Mice were implanted subcutaneously with 28-day osmotic pumps (Alzet, DURECT Corporation, Cupertino CA) filled with 4.5-ng/h D-OC (n=4) or PBS (n=5) ^10,13^. At the end of the study, the kidneys bearing graft were removed using isoflurane and the animals were euthanized by CO2 technique.

### Immunofluorescent imaging

Mouse kidneys with the human islets graft were fixed in 10% formalin, embedded in paraffin, and sectioned at 5 μm. Immunofluorescent histochemistry was performed as previously described ^6^, using sodium citrate (pH 6) antigen retrieval at 95°C for 20 min and 10 min nuclear permeabilization with 0.1% triton-X-100. Sections were blocked with SNIPER (Biocare Medical, Concord, CA), and incubated with primary antibodies to insulin (mouse, Sigma, 1/2000), FOXO1 (rabbit, Abcam ab39670, 1/100), and/or Pdx1 (rabbit, CST 5679S, 1/400), overnight at 4°C. Appropriately matched fluorescent secondary antibodies (1/500, Invitrogen Alexafluor 555, 488) were applied for 2h in the dark, and counterstained with DAPI (1/500). Every β- (insulin-expressing) cell was counted manually per section. All imaging was performed using confocal microscopy (Fluoview 3000, Olympus Life Science, Tokyo, Japan).

### Statistics

All cell line experiments were conducted in triplicate. Data are shown as mean ± SEM. “n” represents the number of biological replicates. Student’s t-test (for comparison between two groups) or two-way ANOVA (for comparisons of three or more groups and two variants) followed by Fisher’s LSD post hoc pairwise tests. Microscopy data were expressed as % mean ± SEM for nuclear PDX1 and FOXO1 compartment as noted, relative to insulin, per mouse transplanted (n = 4-5). Every cell present was counted manually, with positivity recorded as any red nuclear signal; cells were not graded on brightness of signal intensity. Data was analyzed using unpaired Student’s t-tests comparing PBS versus OC treatment per islet donor. All analysis was performed using GraphPad Prism software (v6, GraphPad, San Diego, CA), and with a significance of p < 0.05.

## Results

### Osteocalcin increases phosphorylation of FOXO1-S256.

**Fig. 1.**
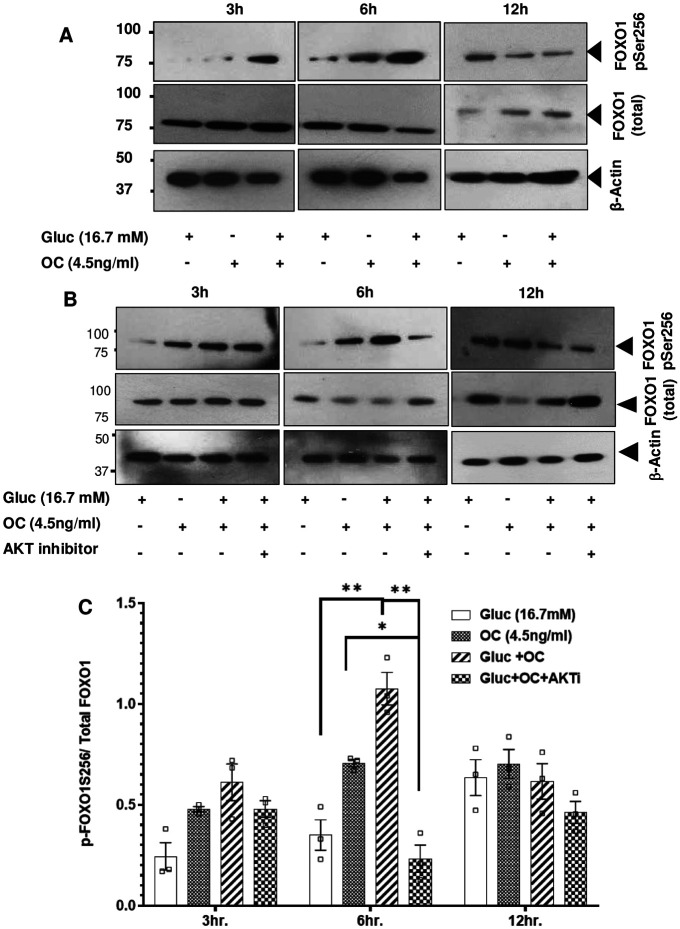
Osteocalcin induces FOXO1 phosphorylation in 1.2B4 β-cells in an AKT-dependent manner. (A) OC-induced FOXO1 phosphorylation in high glucose (16.7mM) with a peak at 6 h as determined by western blot analysis; (B &C) inhibition of AKT results in reduced FOXO1 phosphorylation at serine256. Data are presented as mean ± SEM. Analysis was two-way ANOVA followed by Tukey's multiple comparisons test.

To explore the effect of OC on the phosphorylation of FOXO1-S256, we examined human β-cell line 1.2B4 cultured at high glucose with or without OC. The data show that OC treatment (4.5 ng/ml) in the presence of high glucose (16.7mM) increased the amount of phosphorylated FOXO1-S256 at 6 hr. (Fig. 1A). Furthermore, this increase in FOXO1-S256 at 6h was accompanied by an increase in the phosphorylation of AKT versus total AKT levels when exposed to OC and high glucose (Fig. S1). Moreover, concomitant use of the AKT inhibitor resulted in a significant decrease in FOXO1-S256 phosphorylation (Fig. 1B & C), supporting our observation that OC regulates the transcriptional activity of FOXO1 via the AKT pathway.

### FOXO1 expression is downregulated by osteocalcin following phosphorylation.

**Fig. 2.**
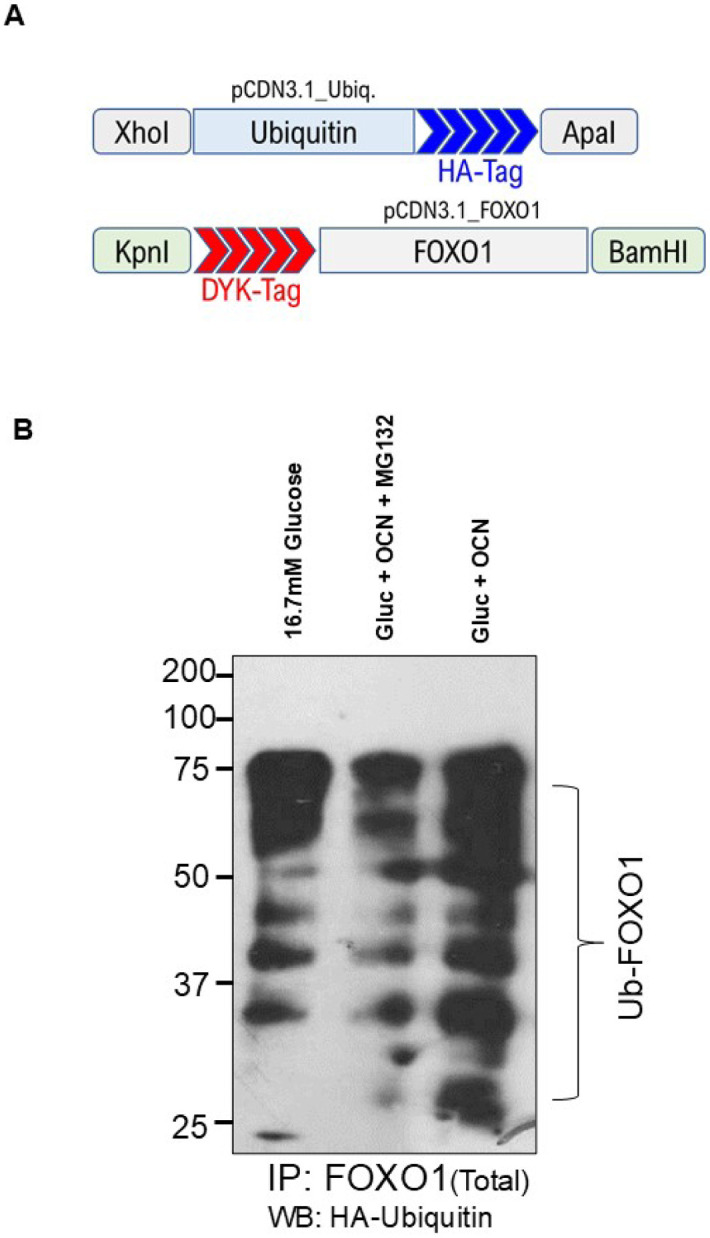
FOXO1 is ubiquitinated after OC treatment in HEK293 cells. (A) HEK293 cells were transfected with DYK-Tag FOXO1 and HA-Tag ubiquitin. (B) HEK293 cells were treated with 16.7mM glucose; glucose, OC, and the proteasomal inhibitor MG132; or glucose +OC only. The ubiquitinated FOXO1 complex was resolved by a 10% SDS-PAGE, followed by western blot with HA antibody.

To study the fate of FOXO1 following phosphorylation in response to treatment with OC, we set an *in vitro* ubiquitination assay of FOXO1. We transfected HEK293 cells with the HA-tag ubiquitin and DYK-tagged FOXO1 (Fig 2A). Co-transfected HEK293 cells were cultured in high glucose (16.7 mM) under 3 conditions: control (high glucose alone), high glucose + OC, or high glucose + OC + proteasomal inhibitor MG132 for 12h and DYK-FOXO1 was immunoprecipitated from harvested cells. As shown in figure 2B, high glucose by itself failed to show any ubiquitination of FOXO1 detection by enhancement of the HA-tag antibody signal, while OC with high glucose induced ubiquitination, and was consequently inhibited by the proteasomal inhibitor MG132. These results suggest that OC directly induced phosphorylation of FOXO1, which was destabilized and degraded through ubiquitination. These data further suggest that FOXO1 degradation was mediated by the proteasomal pathway in OC-treated cells.

### Osteocalcin restores PDX1 expression following phosphorylation-dependent ubiquitination and degradation of FOXO1

**Fig. 3.**
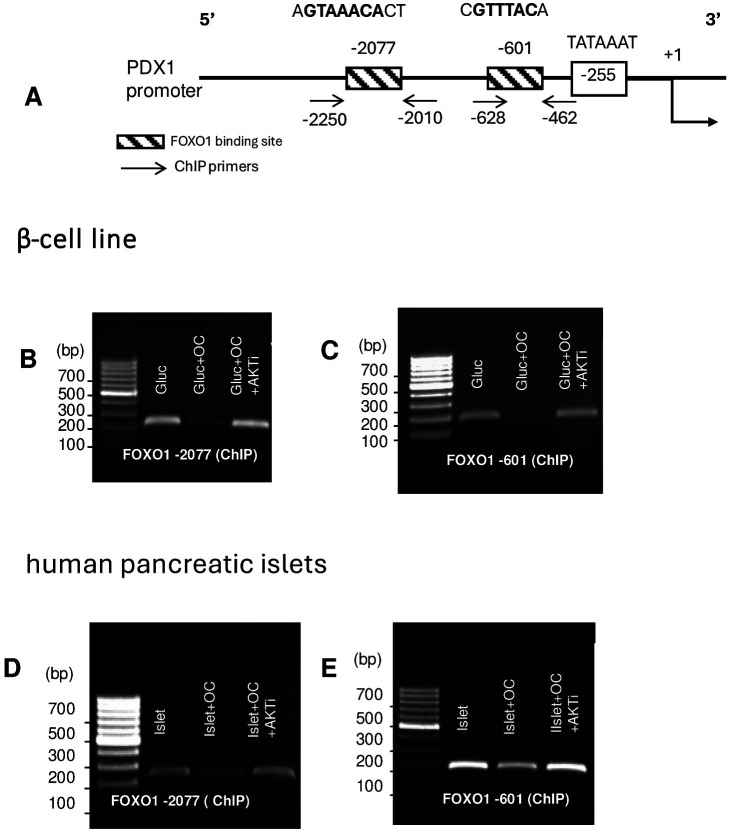
Osteocalcin induces the loss of FOXO1 binding to the PDX1 promoter in a β-cell line and human pancreatic islets. (A) Schematic representation of human PDX1 promoter showing the FOXO1 binding sites and the location of the primers used to determine FOXO1 binding. The TATA box is shown, and all sites are numbered with respect to +1 start site of the PDX1 mRNA. 1.2B4 β-cells were treated with 16.7 mM glucose, glucose + OC, or glucose + OC + AKT inhibitor (AKTi), and the proportion of bound FOXO1-PDX1 promoter was determined by the ChIP assay. (B & C) PCR amplified products of immunoprecipitated DNA by FOXO1 antibody at −2077bp and −601bp site amplicons. (D & E) Binding of FOXO1 of human pancreatic islets analyzed by PCR on immunoprecipitated DNA at −2077bp and −601bp treated with OC, or +OC + AKTi compared to control.

Our finding of the effect of OC on FOXO1 phosphorylation and degradation prompted us to study the effect of OC treatment on FOXO1 binding to the PDX1 promoter. We designed primers for two ChIP sites, at −601 and −2077 using 1.2B4 cells. The −2077 bp site has previously been characterized as FOXO1 binding in the PDX1 promoter ^3^. We added the −601 bp site, given that it is very close to the TATA site at −255 bp and therefore may have some influence in regulating transcription of the PDX1 gene. A schematic representation of the human PDX1 promoter showing the FOXO1 binding sites and the location of the primers used to determine FOXO1 binding is shown in figure 3A.

Serum-starved 1.2B4 cells were cultured for 12h under 3 conditions: 16.7mM glucose alone, glucose + OC (4.5 ng), or glucose+ OC+ AKT inhibitor followed by the ChIP binding assay ^32,33^. There was a significant decrease in FOXO1 precipitation in the presence of OC in both binding sites, which was completely reversed in the presence of the AKT inhibitor (Fig. 3B, −2077 bp; C, −601 bp).

**Fig. 4.**
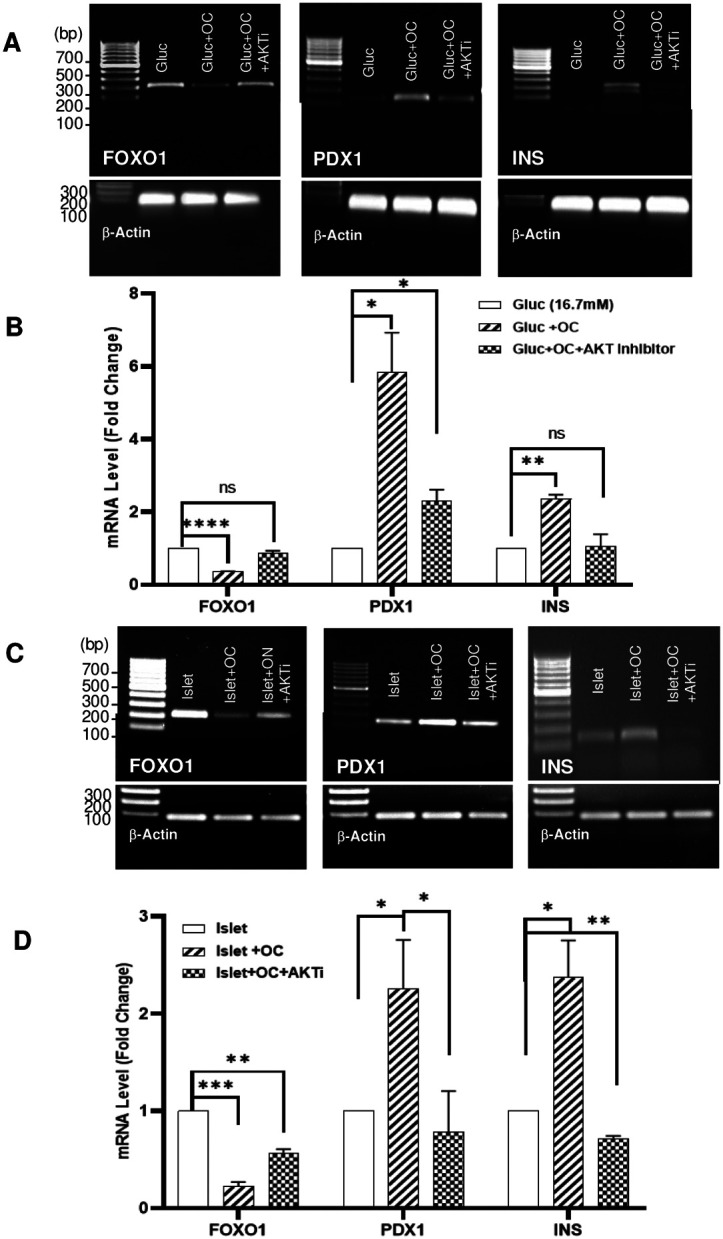
Gene expression changes in FOXO1, PDX1, and insulin from osteocalcin in β-cells. (A & B) Quantitative PCR of 1.2B4 cells treated with 16.7 mM glucose, glucose + OC, or glucose + OC + AKTi depicting gene expression changes in FOXO1, PDX1, insulin, and cDNA. (C & D) Total mRNA expression of FOXO1, PDX1, and insulin in human islets from a high BMI islet donor without diabetes, cultured with OC or OC + AKT inhibitor compared to control. Data are presented as mean ± SEM. Analysis was two-way ANOVA followed by Tukey's multiple comparisons test.

To investigate the direct relationship between FOXO1, insulin, and PDX1, we also performed a ChIP binding assay using both FOXO1 binding sites utilizing human islets. Human islets isolated from a high BMI, non-diabetic donor (Table 1) was similarly treated with high glucose, OC (4.5 ng/ml), and/or 10 μM AKT inhibitor for 7 days. The ChIP assay confirmed results shown in the 1.2B4 cell line, specifically showing a visible decrease in FOXO1 immuno-precipitation at both binding sites in the presence of OC compared to control, and a preservation of FOXO1 binding in those cells treated with OC and AKT inhibitor (Fig. 3D, −2077bp site, E −601 bp site), supporting our earlier observation that OC regulates the transcriptional activity of FOXO1 via the AKT pathway. To gain mechanistic insights into whether OC could restore pancreatic gene expression of insulin and PDX1 under conditions of hyperglycemia, we further examined the gene expression of FOXO1, PDX1 and insulin in β-cells cultured with OC, with or without the AKT inhibitor. High glucose + OC demonstrated a decrease in FOXO1 and an increase in PDX1 and insulin signal in 1.2B4 cells, whereas the addition of AKT inhibitor nullified these effects (Fig. 4A & B). Moreover, RNA isolated from the human islet sample also demonstrated the loss of FOXO1 and induction of PDX1 and insulin when treated with OC, which was abrogated by the AKT inhibitor (Fig. 4C & D). These findings indicate that OC restores the expression of PDX1 and insulin in human β-cells under hyperglycemia through its phosphorylation and degradation of FOXO1 via the AKT pathway.

### Osteocalcin reduces nuclear FOXO1 and drives nuclear PDX1 cellular localization in human islets

**Fig. 5.**
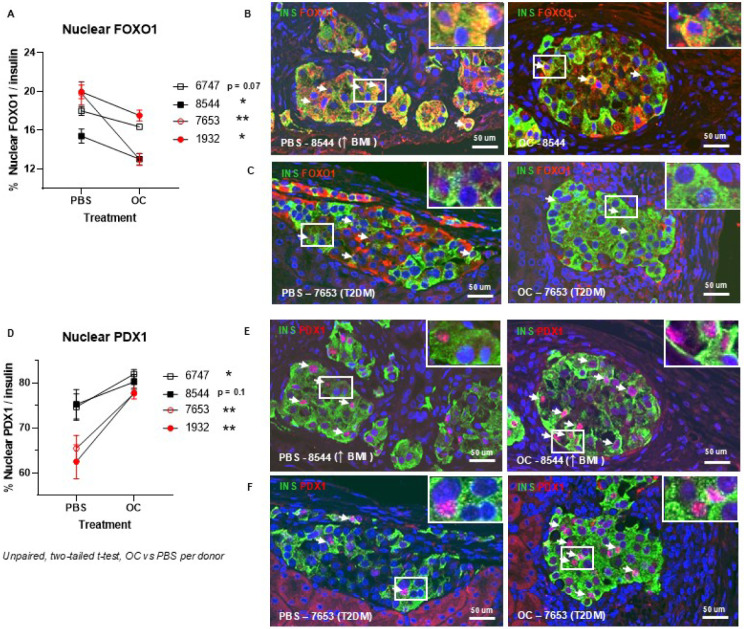
Osteocalcin improves human β-cell identity by FOXO1 and PDX1 nuclear shuttling. Human islets from four high BMI donors, with and without T2DM, showed improvements in β-cell-specific transcription factor proportion in insulin-expressing β-cells (A, D) when exposed to OC (A, D; B, C, E, F, right) relative to PBS-treated controls (A, D; B, C, E, F, left). Islet grafts from kidney capsule sections immunostained for FOXO1 (B, C, red) or PDX1 (E, F, red) relative to insulin (B, C, E, F green) showed a significant decrease in the proportion of nuclear FOXO1 (A) and significant increase in the proportion of nuclear PDX1 (C) in insulin-expressing β-cells, relative to PBS-treatment. Representative images shown are from high BMI donor #8544 (A, B, D, E, solid black squares), and from T2DM donor #7653 (A, C, D, F, open red circles). Data represents unpaired, two-tailed t-tests of the mean of β-cell-transcription factor proportion when treated with OC versus PBS per islet donor; * p <0.05, ** p<0.01. Size bar denotes 50 μm. Arrows indicate cells of interest. A model for FOXO1 regulation through Osteocalcin-induced and phosphorylation-dependent degradation.

The translocation of FOXO1 between the nucleus and cytoplasm in β-cells has been shown to be regulated through the PI3-kinase/AKT signaling cascade ^35,36^, and FOXO1 and PDX1 exhibit mutually exclusive patterns of nuclear localization in β cells, such that mature and functional beta cells exhibit nuclear PDX1 and cytoplasmic localization of FOXO1 ^3^. To determine if OC induces shuttling of FOXO1 between nuclear and cytoplasmic compartments in the human islet, and to support cell-line evidence provided above, isolated islets from high BMI donors (BMI ≥30 mg/kg^2^), two without diabetes and two with diabetes (Table 1) were cultured with and without OC (4.5 ng/ml) for 7 days, then transplanted into mice for 4 weeks ± OC pumps. High BMI donors were chosen for study due to their known high-risk status for alterations in islet identity, even without clinical manifestations of glycemic dysfunction ^37,38^, and comparisons of islets from donors with and without diabetes were done to assess the role of OC in islet phenotypes of overt glycemic dysregulation. Immunostaining of insulin (green, Fig. 5B, C) and FOXO1 (red Fig. 5B, C) exhibited a reduced proportion of nuclear FOXO1 in the β-cell in OC-treated islets relative to PBS-treated cells (Fig. 5A). It should be noted that FOXO1 is also found in pancreatic alpha cells ^39^, shown in figure 5B and C (bright red cells); these cells were not quantified. There was also a significantly increased proportion of nuclear PDX1 (red, Fig. 5E, F) in OC-treated β-cells (green, insulin, Fig. 5E, F) relative to PBS-treated islets from each donor (Figure 5D). These trends were similar, but more exaggerated, in islet donors with T2DM than high BMI islet samples (Fig. 5A, D; red circles denote T2DM; representative images from donor 7653, open red circles, Fig. 5A, C; D, F). There was a higher proportion of nuclear FOXO1, and lower proportion of nuclear PDX1, in PBS-treated, islet donor samples with T2DM than in high BMI samples, whilst OC exposure effected a greater statistical change versus PBS treatment relative to high BMI islet donor samples as well, particularly in the proportional change of nuclear PDX1 (Fig. 5A, D). These improvements in transcription factor presence and subcellular location demonstrate a direct proof-of-principle concept of the importance of osteocalcin signaling in pancreatic β-cell physiology.

## Discussion

**Figure 6.**
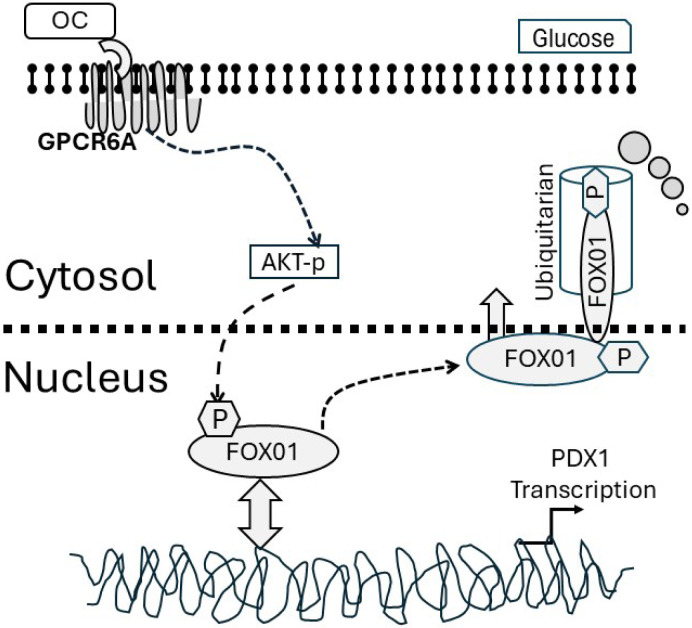


The loss of FOXO1 and PDX1 transcription factor signaling in pancreatic β-cells, which results in the loss of insulin production prevalent in pathological conditions such as insulin resistance and T2DM, has been broadly demonstrated and identified as a key clinical target ^1,40,41^. Here for the first time, we have shown the degradation of FOXO1, phosphorylated by the PI3K-AKT pathway in response to the bone hormone osteocalcin (OC) through the ubiquitin-proteasome system, restores PDX-1 and insulin expression under hyperglycemic conditions, and which is similar to other previously identified FOXO family degradation pathways ^42^. Matsuzaki et al found that the degradation of FOXO phosphorylated by the PI3K-PKB pathway in response to insulin requires both phosphorylation and cytoplasmic retention ^42^. Based on this finding, we report a mechanism for the regulation of PDX1 and insulin in pancreatic cells by OC, such that when OC binds to its specific receptor GPCR6A ^26^, it activates the downstream signaling cascade: FOXO1 is phosphorylated by AKT and consequently excluded from the nucleus (Fig. 6). Phosphorylated FoxO1 is then ubiquitinated in the cytoplasm, followed by degradation, thus restoring PDX1 and insulin expression.

The loss of β-cell identity, including the failure to express pancreatic β-cell transcription factors (e.g., FOXO1 and PDX1) which directly regulate the transcription of insulin, is associated with loss of β-cell function. Studies of specimens from patients with T2DM or pancreatitis suggest that the loss of β-cell identity could be a result of dedifferentiation which contributes to diabetes progression in humans ^43,44^. Moreover, the increase in hormone-negative endocrine cells shown in patients with diabetes compared to non-diabetic controls is accompanied by a decrease in the number of insulin-positive cells expressing the transcription factor FOXO1 ^45^. An inhibitor-mediated β-cell dedifferentiation model reveals distinct roles for FOXO1 in glucagon repression and insulin maturation ^40,46^. Our data shown here, in both a human beta cell line (1.2B4) and human islets representative of variable disease phenotypes, provides complementary information about how OC functions in conditions of hyperglycemia. The importance of testing this process in diverse samples permits a more in-depth study of our hypothesis due to the testing limits of using either cell lines and human samples alone, and demonstrates that a restoration of islet identity is accomplished by appropriate cell signaling pathways as regulated by OC.

The transcriptional activity of the FOXO family is regulated by different post-translational modifications such as phosphorylation, acetylation, and ubiquitination, which affect their subcellular localization, binding with other regulatory factors, and degradation. One of the basic upstream regulatory inputs is the kinase AKT ^47^, which induces phosphorylation of FOXO transcription factors. Phosphorylated FOXO proteins are negatively regulated by nuclear exclusion ^48,49^. When AKT is in an inactivated state, FOXO proteins stay in the nucleus and regulate target gene expression. Our data demonstrates the correlation between OC treatment and the phosphorylation of FOXO1 at S256 via the PI3K-PKB pathway, and that inhibiting AKT effected phosphorylation and hence degradation of FOXO1. Kitamura et al. reported that FOXO1 and FOXA2 shared common DNA-binding sites in the PDX1 promoter ^1^. Here we have shown evidence supporting this pathway in direct cell line signaling experiments and in human islets exposed to osteocalcin and suggests a critical and fundamental role of the OC hormone in PDX1 expression and insulin transcription. While osteocalcin is not yet available as an adjunct pharmaceutical agent for metabo-glycemic regulation, including T2DM, it is being actively investigated in clinical trials ^50^; our data here strongly supports the continuing evaluation of this hormone in improving insulin action and restoring beta cell function under conditions of metabolic stress by proposing a mechanism of action.

## Figures and Tables

**Table 1.** Human islet donor characteristics

RRID	Sex	Age (years)	B (kg/m^2^)	HbA1c (%)	Race	Diagnosed T2DM
Sample used for ChIP assay and mRNA analysis						
SAMN32641505	M	16	29.5	5.5	H/L	No
Samples used for microscopy						
SAMN15656747	M	24	33.6	5.1	W	No
SAMN11578544	F	44	38.7	5.9	H/L	No
SAMN12597653	F	42	40.3	9.5	H/L	Yes
SAMN 17831932	M	60	41.3	7.5	H/L	Yes

Race: W = white; H/L = Hispanic/ Latino. M = male; F = female. T2DM = type 2 diabetes mellitus.

**Table 2.** Primer sequences designed for cDNA gene expression and ChIP assay of human pancreatic β-cells.

Gene	NM number	Forward (5’)	Reverse (3’)
*cDNA gene expression primers*			
**Actin**	NM_001101.3	AGCACAGAGCCTCGCCTT	CATCATCCATGGTGAGCTGG
**PDX1**	NM_000209	GAAGTCTACCAAAGCTCACGCG	GGAACTCCTTCTCCAGCTCTAG
**FOXO1**	NM_002015	AAGAGCGTGCCCTACTTCAA	GTTGTTGTCCATGGATGCAG
**Insulin**	NM_000207.1	CCCTGCAGAAGCGTGGCATT	CCATCTCTCTCGGTGCAGGA
*FOXO1 ChIP site on PDX1 promoter primers*			
**−2077**	NM_00209.4	CGGATTTGCTTTCTGCTGAG	GCACCCCTACACCCACTCT
**−601**	NM_00209.4	GTCTCTGTGAATGCTTCAGA	TTGCAGACCCAGCTCTCTAG

## Data Availability

The authors declare that the data supporting the findings of this study are available within the paper and its Supplementary Information files. Should any raw data files be needed in another format they are available from the corresponding author upon reasonable request.

## References

[1] Kitamura T. The role of FOXO1 in beta-cell failure and type 2 diabetes mellitus. Nat Rev Endocrinol 9, 615–623 (2013). 10.1038/nrendo.2013.157 23959366

[2] Buteau J. & Accili D. Regulation of pancreatic beta-cell function by the forkhead protein FoxO1. Diabetes Obes Metab 9 Suppl 2, 140–146 (2007). 10.1111/j.1463-1326.2007.00782.x 17919188

[3] Kitamura T. The forkhead transcription factor Foxo1 links insulin signaling to Pdx1 regulation of pancreatic beta cell growth. J Clin Invest 110, 1839–1847 (2002). 10.1172/JCI16857 12488434 PMC151657

[4] Kaneto H. PDX-1 functions as a master factor in the pancreas. Front Biosci 13, 6406–6420 (2008). 10.2741/3162 18508668

[5] Gao T. Pdx1 maintains beta cell identity and function by repressing an alpha cell program. Cell Metab 19, 259–271 (2014). 10.1016/j.cmet.2013.12.002 24506867 PMC3950964

[6] Beamish C. A. Variability in endocrine cell identity in patients with chronic pancreatitis undergoing islet autotransplantation. Am J Transplant 19, 1568–1576 (2019). 10.1111/ajt.15154 30372580

[7] White M. G. Expression of mesenchymal and alpha-cell phenotypic markers in islet beta-cells in recently diagnosed diabetes. Diabetes Care 36, 3818–3820 (2013). 10.2337/dc13-0705 24062329 PMC3816907

[8] Marselli L. Are we overestimating the loss of beta cells in type 2 diabetes? Diabetologia 57, 362–365 (2014). 10.1007/s00125-013-3098-3 24233056

[9] Lee N. K. Endocrine regulation of energy metabolism by the skeleton. Cell 130, 456–469 (2007). 10.1016/j.cell.2007.05.047 17693256 PMC2013746

[10] Beamish C. A. Osteocalcin protects islet identity in low-density lipoprotein receptor knockout mice on high-fat diet. J Endocrinol 261 (2024). 10.1530/JOE-23-0352 38305305

[11] Ferron M., Hinoi E., Karsenty G. & Ducy P. Osteocalcin differentially regulates beta cell and adipocyte gene expression and affects the development of metabolic diseases in wild-type mice. Proc Natl Acad Sci U S A 105, 5266–5270 (2008). 10.1073/pnas.0711119105 18362359 PMC2278202

[12] Gupte A. A. Osteocalcin protects against nonalcoholic steatohepatitis in a mouse model of metabolic syndrome. Endocrinology 155, 4697–4705 (2014). 10.1210/en.2014-1430 25279794 PMC5393336

[13] Sabek O. M. Osteocalcin Effect on Human beta-Cells Mass and Function. Endocrinology 156, 3137–3146 (2015). 10.1210/EN.2015-1143 26151356

[14] Zhou B. Osteocalcin reverses endoplasmic reticulum stress and improves impaired insulin sensitivity secondary to diet-induced obesity through nuclear factor-kappaB signaling pathway. Endocrinology 154, 1055–1068 (2013). 10.1210/en.2012-2144 23407450

[15] Lacombe J. Measurement of bioactive osteocalcin in humans using a novel immunoassay reveals association with glucose metabolism and beta-cell function. Am J Physiol Endocrinol Metab 318, E381–E391 (2020). 10.1152/ajpendo.00321.2019 31935114 PMC7395472

[16] Zhou M. Serum osteocalcin concentrations in relation to glucose and lipid metabolism in Chinese individuals. Eur J Endocrinol 161, 723–729 (2009). 10.1530/EJE-09-0585 19671707

[17] Polgreen L. E. Association of osteocalcin with obesity, insulin resistance, and cardiovascular risk factors in young adults. Obesity (Silver Spring) 20, 2194–2201 (2012). 10.1038/oby.2012.108 22573135 PMC3483437

[18] Fernandez-Real J. M. The relationship of serum osteocalcin concentration to insulin secretion, sensitivity, and disposal with hypocaloric diet and resistance training. J Clin Endocrinol Metab 94, 237–245 (2009). 10.1210/jc.2008-0270 18854399

[19] Kanazawa I. Serum osteocalcin/bone-specific alkaline phosphatase ratio is a predictor for the presence of vertebral fractures in men with type 2 diabetes. Calcif Tissue Int 85, 228–234 (2009). 10.1007/s00223-009-9272-4 19641839

[20] Pittas A. G., Harris S. S., Eliades M., Stark P. & Dawson-Hughes B. Association between serum osteocalcin and markers of metabolic phenotype. J Clin Endocrinol Metab 94, 827–832 (2009). 10.1210/jc.2008-1422 19088165 PMC2681283

[21] Shea M. K. Gamma-carboxylation of osteocalcin and insulin resistance in older men and women. Am J Clin Nutr 90, 1230–1235 (2009). 10.3945/ajcn.2009.28151 19776145 PMC2762158

[22] Yeap B. B. Higher serum undercarboxylated osteocalcin and other bone turnover markers are associated with reduced diabetes risk and lower estradiol concentrations in older men. J Clin Endocrinol Metab 100, 63–71 (2015). 10.1210/jc.2014-3019 25365314

[23] Ferron M., McKee M. D., Levine R. L., Ducy P. & Karsenty G. Intermittent injections of osteocalcin improve glucose metabolism and prevent type 2 diabetes in mice. Bone 50, 568–575 (2012). 10.1016/j.bone.2011.04.017 21550430 PMC3181267

[24] Pi M. Role of GPRC6A in Regulating Hepatic Energy Metabolism in Mice. Sci Rep 10, 7216 (2020). 10.1038/s41598-020-64384-8 32350388 PMC7190669

[25] Wei J., Hanna T., Suda N., Karsenty G. & Ducy P. Osteocalcin promotes beta-cell proliferation during development and adulthood through Gprc6a. Diabetes 63, 1021–1031 (2014). 10.2337/db13-0887 24009262 PMC3931403

[26] Pi M. Evidence for Osteocalcin Binding and Activation of GPRC6A in beta-Cells. Endocrinology 157, 1866–1880 (2016). 10.1210/en.2015-2010 27007074 PMC4870875

[27] Pi M., Nishimoto S. K. & Quarles L. D. GPRC6A: Jack of all metabolism (or master of none). Mol Metab 6, 185–193 (2017). 10.1016/j.molmet.2016.12.006 28180060 PMC5279936

[28] Pi M. & Quarles L. D. Multiligand specificity and wide tissue expression of GPRC6A reveals new endocrine networks. Endocrinology 153, 2062–2069 (2012). 10.1210/en.2011-2117 22374969 PMC3339644

[29] Pi M., Wu Y. & Quarles L. D. GPRC6A mediates responses to osteocalcin in beta-cells in vitro and pancreas in vivo. J Bone Miner Res 26, 1680–1683 (2011). 10.1002/jbmr.390 21425331 PMC5079536

[30] Fraga D. W., Sabek O., Hathaway D. K. & Gaber A. O. A comparison of media supplement methods for the extended culture of human islet tissue. Transplantation 65, 1060–1066 (1998). 10.1097/00007890-199804270-00009 9583866

[31] Sarkar S., Kantara C. & Singh P. Clathrin mediates endocytosis of progastrin and activates MAPKs: role of cell surface annexin A2. Am J Physiol Gastrointest Liver Physiol 302, G712–722 (2012). 10.1152/ajpgi.00406.2011 22241862 PMC3330782

[32] O'Connell M. R. Epigenetic changes and alternate promoter usage by human colon cancers for expressing DCLK1-isoforms: Clinical Implications. Sci Rep 5, 14983 (2015). 10.1038/srep14983 26447334 PMC4597220

[33] Sarkar S. FOXD3 Regulates CSC Marker, DCLK1-S, and Invasive Potential: Prognostic Implications in Colon Cancer. Mol Cancer Res 15, 1678–1691 (2017). 10.1158/1541-7786.MCR-17-0287 28851816 PMC5748292

[34] Ishizawa M., Kobayashi Y., Miyamura T. & Matsuura S. Simple procedure of DNA isolation from human serum. Nucleic Acids Res 19, 5792 (1991). 10.1093/nar/19.20.5792 1945860 PMC328998

[35] Eijkelenboom A. & Burgering B. M. FOXOs: signalling integrators for homeostasis maintenance. Nat Rev Mol cell Biol 14, 83–97 (2013). 10.1038/nrm3507 23325358

[36] Martinez S. C., Cras-Meneur C., Bernal-Mizrachi E. & Permutt M. A. Glucose regulates Foxo1 through insulin receptor signaling in the pancreatic islet beta-cell. Diabetes 55, 1581–1591 (2006). 10.2337/db05-0678 16731820

[37] Atanes P., Ashik T. & Persaud S. J. Obesity-induced changes in human islet G protein-coupled receptor expression: Implications for metabolic regulation. Pharmacol Ther 228, 107928 (2021). 10.1016/j.pharmthera.2021.107928 34174278

[38] Boye K. S. Obesity and glycemic control among people with type 2 diabetes in the United States: A retrospective cohort study using insurance claims data. J Diabetes Complications 35, 107975 (2021). 10.1016/j.jdiacomp.2021.107975 34176723

[39] McKinnon C. M., Ravier M. A. & Rutter G. A. FoxO1 is required for the regulation of preproglucagon gene expression by insulin in pancreatic alphaTC1-9 cells. J Biol Chem 281, 39358–39369 (2006). 10.1074/jbc.M605022200 17062568

[40] Talchai C., Xuan S., Lin H. V., Sussel L. & Accili D. Pancreatic beta cell dedifferentiation as a mechanism of diabetic beta cell failure. Cell 150, 1223–1234 (2012). 10.1016/j.cell.2012.07.029 22980982 PMC3445031

[41] Teaney N. A. & Cyr N. E. FoxO1 as a tissue-specific therapeutic target for type 2 diabetes. Front Endocrinol (Lausanne) 14, 1286838 (2023). 10.3389/fendo.2023.1286838 37941908 PMC10629996

[42] Matsuzaki H., Daitoku H., Hatta M., Tanaka K. & Fukamizu A. Insulin-induced phosphorylation of FKHR (Foxo1) targets to proteasomal degradation. Proc Natl Acad Sci U S A 100, 11285–11290 (2003). 10.1073/pnas.1934283100 13679577 PMC208749

[43] Amo-Shiinoki K. Islet cell dedifferentiation is a pathologic mechanism of long-standing progression of type 2 diabetes. JCI Insight 6 (2021). 10.1172/jci.insight.143791 PMC7821596 33427207

[44] Hunter C. S. & Stein R. W. Evidence for Loss in Identity, De-Differentiation, and Trans-Differentiation of Islet beta-Cells in Type 2 Diabetes. Front Genet 8, 35 (2017). 10.3389/fgene.2017.00035 28424732 PMC5372778

[45] Efrat S. Beta-Cell Dedifferentiation in Type 2 Diabetes: Concise Review. Stem cells 37, 1267–1272 (2019). 10.1002/stem.3059 31298804

[46] Accili D. When beta-cells fail: lessons from dedifferentiation. Diabetes Obes Metab 18 Suppl 1, 117–122 (2016). 10.1111/dom.12723 27615140 PMC5021187

[47] Boccitto M. & Kalb R. G. Regulation of Foxo-dependent transcription by post-translational modifications. Curr Drug Targets 12, 1303–1310 (2011). 10.2174/138945011796150316 21443461 PMC3794852

[48] Biggs W. H. 3rd, Meisenhelder J., Hunter T., Cavenee W. K. & Arden K. C. Protein kinase B/Akt-mediated phosphorylation promotes nuclear exclusion of the winged helix transcription factor FKHR1. Proc Natl Acad Sci U S A 96, 7421–7426 (1999). 10.1073/pnas.96.13.7421 10377430 PMC22101

[49] Brunet A. Akt promotes cell survival by phosphorylating and inhibiting a Forkhead transcription factor. Cell 96, 857–868 (1999). 10.1016/s0092-8674(00)80595-4 10102273

[50] Liu Y. Relationship between serum osteocalcin/undercarboxylated osteocalcin and type 2 diabetes: a systematic review/meta-analysis study protocol. BMJ Open 9, e023918 (2019). 10.1136/bmjopen-2018-023918 PMC6429918 30862632

